# Life worth living: cross-sectional study on the prevalence and determinants of the wish to die in elderly patients hospitalized in an internal medicine ward

**DOI:** 10.1186/s12877-020-01762-x

**Published:** 2020-09-14

**Authors:** Marc-Antoine Bornet, Eve Rubli Truchard, Gérard Waeber, Peter Vollenweider, Mathieu Bernard, Laure Schmied, Pedro Marques-Vidal

**Affiliations:** 1 Service of Internal Medicine, Lausanne University Hospital, Rue du Bugnon 46, 1011 Lausanne, Switzerland; 2 Chair of Geriatric Palliative Care, Lausanne University Hospital, Lausanne, Switzerland; 3 Service of Palliative and Supportive Care, Lausanne University Hospital, Lausanne, Switzerland

**Keywords:** Wish to die, Quality of life, Acute care, Internal medicine, Switzerland

## Abstract

**Background:**

Elderly people frequently express the wish to die: this ranges from a simple wish for a natural death to a more explicit request for death. The frequency of the wish to die and its associated factors have not been assessed in acute hospitalization settings. This study aimed to investigate the prevalence and determinants of the wish to die in elderly (≥65 years) patients hospitalized in an internal medicine ward.

**Methods:**

This cross-sectional study was conducted between 1 May, 2018, and 30 April, 2019, in an acute care internal medicine ward in a Swiss university hospital. Participants were a consecutive sample of 232 patients (44.8% women, 79.3 ± 8.1 years) with no cognitive impairment. Wish to die was assessed using the Schedule of Attitudes toward Hastened Death-senior and the Categories of Attitudes toward Death Occurrence scales.

**Results:**

Prevalence of the wish to die was 8.6% (95% confidence interval [CI]: 5.3–13.0). Bivariate analysis showed that patients expressing the wish to die were older (*P* = .014), had a lower quality of life (*P* < .001), and showed more depressive symptoms (*P* = .044). Multivariable analysis showed that increased age was positively (odds ratio [OR] for a 5-year increase: 1.43, 95% CI 0.99–2.04, *P* = .048) and quality of life negatively (OR: 0.54, 95% CI 0.39–0.75, *P* < 0.001) associated with the likelihood of wishing to die. Participants did not experience stress during the interview.

**Conclusions:**

Prevalence of the wish to die among elderly patients admitted to an acute hospital setting is low, but highly relevant for clinical practice. Older age increases and better quality of life decreases the likelihood of wishing to die. Discussion of death appears to be well tolerated by patients.

## Background

Elderly people frequently express the wish to die (WTD): this ranges from a simple wish for a natural death to more extreme expressions of an explicit request to die [1]. A Dutch study of 1794 people aged 58 to 98 years showed that 3.4% had wished to die during the previous week, and that 15.3% had reported such thoughts in the past [2]. A Swiss study of 101 patients aged ≥65 years in a geriatric rehabilitation setting showed that up to 14.9% wished to die [3]; another study of 280 nursing home residents found that up to 22.1% wished to die, almost all of who wished for a natural death [4].

Studies of elderly persons have identified a relationship between the WTD and lower social support [2, 5], depression [2, 6, 7], lower religiosity [8], and low self-assessed health [9]. A review of clinical studies showed that non-physical determinants (psychological, spiritual, and social dimensions) are the most important causes of the WTD [7]. Conversely, quality of life (QoL) may be an understudied and important determinant of the WTD, given the substantial literature indicating an association between suicide and decreased QoL [10, 11].

Patients admitted to internal medicine units frequently present with multimorbidity [12], which can degrade QoL and hasten the WTD. This multimorbidity requires complex diagnostic and therapeutic strategies. Development of a therapeutic plan is particularly challenging when the patient wishes to die. Moreover, no studies have explored the WTD in this acute setting, and the issue is not part of clinical routine. By contrast, attitudes to resuscitation are commonly investigated [13]. A WTD may be indirectly expressed if the patient chooses a do-not-resuscitate (DNR) order.

Hence, this study aimed to assess the prevalence, determinants, and consequences for DNR orders of the WTD among patients aged ≥65 years admitted to an internal medicine ward. Our hypotheses were that the WTD is frequently expressed in patients hospitalized in internal medicine, and that the patient’s QoL, and social, psychological, spiritual, and biological factors influence the WTD and the attitude toward DNR orders. This study also aimed to assess the degree of stress generated in the interviewee by an interview querying their WTD attitude.

## Methods

### Setting

This cross-sectional study was conducted between 1 May, 2018, and 30 April, 2019, in the internal medicine ward of Lausanne University Hospital, Switzerland. The internal medicine ward has a 166-bed capacity and admits 5550 patients per year, two-thirds of who are ≥65 years old.

### Sample size

Sample size was based on data reported by Rurup et al. [2] and calculations were performed using PS Power and Sample Size version 3.0 [14]. Considering a WTD prevalence of 15% [2], an alpha value of 5%, and a power of 80%, a sample size of 250 was considered adequate. Considering a 50% exclusion rate, a 40% refusal rate, and 10% of the records to have missing data, it was necessary to screen 900 patients.

### Selection procedure

Before being considered eligible for the study, patients aged ≥65 years were screened for language skills and cognitive impairment. For pragmatic reasons, screening was conducted on the second day of hospitalization; the first day was too busy owing to all the clinical procedures performed during the admission process. As a large number of patients were admitted to the ward, it was not possible to screen them all. The study nurse visited a different sector each day according to a previously established schedule. All patients within the selected sector were screened. Patients were excluded from this screening if one of the following conditions was present: 1) previous participation in the study; 2) refusal of cognitive screening; and 3) cognitive screening not feasible (e.g. patient transferred to another unit or too ill to cooperate). Cognitive evaluation was performed using the 6-Item Cognitive Screener [15] and cognitive impairment was defined as a score < 3. Patients unable to speak French or who had cognitive impairment were considered ineligible to participate. Patients eligible for participation were invited to participate and given written information about the study. In accordance with the relevant Swiss legislation and ethics committee guidelines, eligible patients had a 24-h period to make their decision about participating; after this 24-h period, patients willing to participate signed a consent form and were included in the study.

### Data collection

Data were collected in face-to-face interviews with a research nurse trained in palliative care. Participants completed several standardised study questionnaires, which have been previously published elsewhere and are free to use for research purposes. These instruments are described below. The interviews lasted approximately 1 h and were conducted in a closed room, where the research nurse was alone with the participant. Special attention was paid to study participants: the nurse listened patiently and carefully to each participant.

### Wish to die

As WTD instruments vary [3], we assessed WTD using two validated instruments to ensure that the construct was adequately measured in this large sample. We considered patients to show a WTD if responses on either or both instruments were positive.

The French version of the Schedule of Attitudes toward Hastened Death-senior (SAHD-senior) assesses the intensity of the WTD in elderly persons by evaluating attitudes to death, the wish to die/live, and the fear of physical/psychological suffering [3]. It is a modified version of the SAHD, which was originally validated for young terminal stage patients [16]. The SAHD-senior consists of 20 true–false statements; the total score ranges between 0 and 20. Scores ≥10 indicate a WTD.

The French version of the Categories of Attitudes toward Death Occurrence (CADO) measures quality of the WTD [3]. It is based on a qualitative study conducted by Schroepfer [17] and contains six categories (1: neither ready nor accepting; 2: not ready but accepting; 3: ready and accepting; 4: ready, accepting, and wishing death would come; 5: considering a hastened death but having no specific plan; and 6: considering a hastened death with a specific plan). Categories 4 to 6 express an active WTD.

### Other covariates

QoL was assessed using two instruments. The first was the French version of the Quality of life – Control, Autonomy, Self-realization and Pleasure (CASP-12) [18], which was specifically developed for elderly respondents [19, 20]. The CASP-12 consists of 12 questions; responses are on a Likert scale of 0 to 3. The total score ranges between 0 and 36; higher scores indicate better QoL. The second instrument was a single question scored on a numerical scale between 0 (‘worst QoL’) and 10 (‘best QoL’). This scale has been shown to be reliable compared with multi-item scales [21, 22].

Social and family support were evaluated using the French version of the Medical Outcomes Study Social Support Survey (MOS-SSS) [23, 24]. This validated tool contains 19 questions that rate four dimensions (tangible support, affective support, positive social interaction, and emotional/informational support). Responses are on a Likert scale ranging from 1 to 5. The pooled responses are converted to a score ranging between 0 and 100; high scores indicate excellent social support.

Depressive symptoms were assessed using the 20-item Center for Epidemiologic Studies-Depression (CES-D) scale [25]. The CES-D has been validated for elderly respondents and translated into French [26]. It comprises 20 questions, and responses are on a 4-point Likert scale. The final score ranges between 0 and 60; scores ≥16 indicate clinical depression.

Spirituality was assessed using three questions (spiritual person, place in life, importance during illness) from the semi-structured clinical interview SPIR (Spiritual needs and preferences) [27]. Responses to each question are on a numerical scale ranging between 0 and 10; higher values indicate that the respondent places great importance on spirituality. The adapted SPIR score was the average of all responses.

The degree of stress generated by an interview querying the WTD attitude was assessed at the end of the interview on a numerical scale between 0 and 10. Higher scores indicate greater stress.

Do-not-resuscitate (DNR) orders are requested from all patients hospitalized at Lausanne University Hospital [13]. This information was collected at admission and recorded in the patient’s electronic record.

Information about age, gender, case severity (Charlson score) [28], number of medications (at admission), and functional status (Combined Braden activity and mobility subscale) [29] were obtained from medical records.

### Ethical statement

The ethics commission of Canton Vaud (CER-VD, www.cer-vd.ch) approved the study (reference 2017–01875, decision of 6 December, 2017). The study was performed in accordance with the Helsinki declaration and its former amendments, and in accordance with the relevant Swiss legislation.

As a safety issue, we systematically monitored if participants needed to begin psychiatric treatment related to the WTD during the first 2 weeks of hospitalization. If over 10% had experienced distress, the study would have been stopped. In the end, no participants presented a worsening of their psychological status following the interview.

### Statistical analysis

Statistical analyses were performed using Stata version 15.1 (Stata Corp, College Station, Texas, USA). Missing data (which were infrequent) were not replaced. WTD prevalence was expressed as percentages and 95% confidence intervals (CI) calculated using the Poisson method. Agreement between the two WTD instruments (SAHD-senior and CADO) was assessed using Cohen’s kappa. Following previous studies [4], data for all patients expressing a WTD on at least one of the two instruments were pooled. Descriptive results for the WTD (yes/no) were expressed as averages ± standard deviations (SD) or as medians [interquartile ranges] for continuous variables, and as number of participants (percentages) for categorical variables.

Bivariate analyses were performed using Student’s t-test or the Mann–Whitney test for continuous variables and the chi-square test for categorical variables. Variables significantly associated with the WTD in a bivariate analysis were then tested using a multivariable analysis, as follows: first, each variable was tested after adjusting for age; second, all variables were included in a single model; third, the results of the second step were confirmed using stepwise backward and forward procedures, with a *P*-value for entry of .05 and a *P*-value for removal of .10. Multivariable analysis was performed using logistic regression and the results were expressed as odds ratios (OR) and 95% CIs. For multivariable analysis, the effects of a 5-year increase in age, a 5-point increase in the CASP-12 score, and a 2-point increase in the CES-D score were used. Statistical significance was assessed at *P* < .05.

## Results

### Participants

**Fig. 1:**
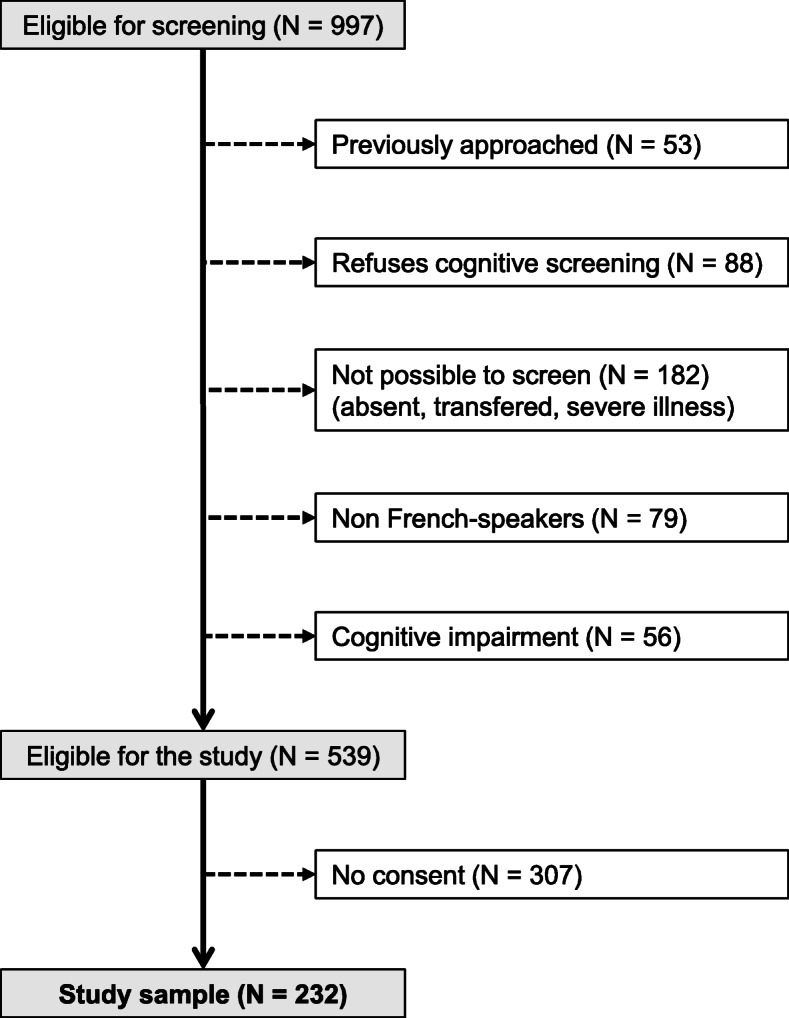
Selection of participants

Of the initial 997 patients eligible for screening, 539 (54%) were eligible for study inclusion and 232 (23%) consented to participate. The selection procedure is summarized in Fig. 1 and the characteristics of patients who did and did not consent are summarized in Additional Table 1. Patients who consented had a lower case severity than patients who did not consent, but no difference was found in other characteristics.

### Prevalence of the wish to die

**Fig. 2:**
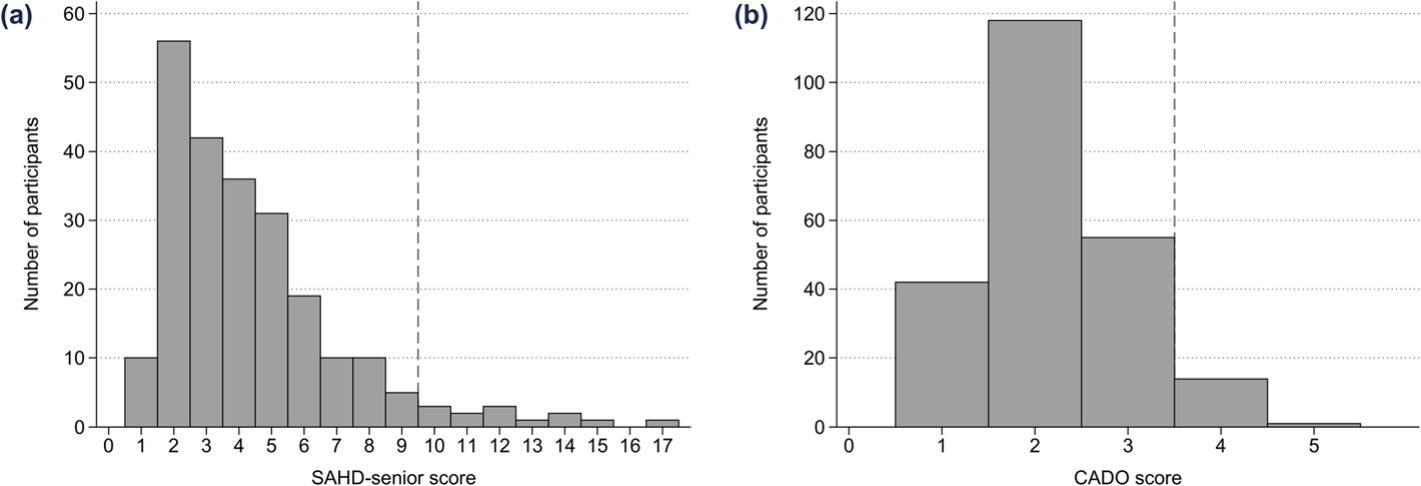
**a** and **b** Distribution of (**a**) SAHD-senior and of (**b**) CADO scores. *SAHD-senior, Schedule of Attitudes toward Hastened Death-senior; CADO, Categories of Attitudes toward Death Occurrence*

The distribution of SAHD-senior and of CADO scores is shown in Figs. 2a and b, respectively. The mean SAHD-senior score was 4.4 (SD 2.8, median 4.0) and the mean CADO score was 2.2 (SD 0.8, median 2.0).

The prevalence (95% CI) of the WTD was 5.6% (3.0–9.4; *N* = 13) according to SAHD-senior scores, 6.5% (3.7–10.4; *N* = 15) according to CADO scores, and 8.6% (5.3–13.0; *N* = 20) for both instruments. The agreement between the SAHD-senior and CADO scores was 0.544 (kappa), *P* < .001.

### Determinants of the wish to die

The characteristics of patients reporting and not reporting a WTD are summarized in Table 1. Compared with patients not wishing to die, patients expressing the WTD were significantly older, had a lower QoL, and more depressive symptoms (Table 1).

**Table 1:** Clinical characteristics of participants, overall and according to the presence of a wish to die

	Total sample	No wish to die	Wish to die	***P***-value
Sample size	232	212	20	
Age, mean (SD), years	79.3 (8.1)	78.9 (8.2)	83.6 (6.4)	.014
Women, %	44.8	44.8	45.0	.987
CASP-12, mean (SD)	26.1 (5.3)	26.5 (5.1)	21.4 (5.3)	<.001
QoL - Single question, mean (SD)	6.9 (1.8)	7.1 (1.6)	4.8 (2.6)	<.001^a^
MOS-SSS, mean (SD)	73.6 (15.6)	74.0 (15.7)	69.2 (14.1)	.114^a^
CES-D, mean (SD)	12.5 (6.7)	12.2 (6.5)	16.0 (7.8)	.044^a^
Adapted SPIR, mean (SD)	5.9 (3.2)	5.9 (3.3)	5.7 (2.7)	.619^a^
Charlson score, mean (SD)	5.9 (11.0)	5.3 (8.1)	12.7 (26.3)	.447^a^
Number of medications, mean (SD)	7.4 (3.8)	7.5 (3.9)	5.9 (3.4)	.067^a^
Functional status, mean (SD)	6.3 (1.3)	6.3 (1.3)	6.2 (1.3)	.805
DNR order, %	71.4	69.8	88.2	.108

*Adapted SPIR* Spiritual needs and preferences (score: 0 to 10), *CASP-12* Control, autonomy, self-realization and pleasure 12 items – French version (score: 0 to 36), *CES-D* Center for epidemiologic studies - depression (score: 0 to 60), *DNR* do not resuscitate, *MOS-SSS* Medical outcome study social support survey (score: 0 to 100), *QoL* Quality of life (score: 0 to 10). Wish to die defined as either a SAHD-senior score ≥ 10 or a CADO category ≥4. Between-group comparisons performed using Student’s t-test or Mann–Whitney test (^a^) for continuous variables and chi-square for categorical variables

The results of the multivariable analysis are presented in Table 2. After adjusting for age, QoL (CASP-12 or single question) was negatively associated with the WTD, whereas depressive symptoms were positively associated. When all variables were included in the model, age was positively associated and QoL (single question) was negatively associated with the WTD. However, there was no association between the WTD and depressive symptoms or QoL as assessed using the CASP-12. These findings were confirmed by the stepwise logistic regression (not shown).

**Table 2:** Multivariable analysis of determinants of the wish to die

	Model 1	***P***-value	Model 2	***P***-value
Age (per 5-year increase)	–		1.43 (1.00–2.04)	.048
CASP-12 (per 5-point increase)	0.41 (0.26–0.66)	<.001	0.84 (0.44–1.60)	.599
QoL - Single question	0.54 (0.41–0.70)	<.001	0.54 (0.39–0.75)	<.001
CES-D (per 2-point increase)	1.17 (1.02–1.33)	.022	1.00 (0.84–1.20)	.984

*CASP-12* Control, autonomy, self-realization and pleasure 12 items – French version, *CES-D* Center for epidemiologic studies – depression, *QoL* Quality of life

Model 1: adjusted for age; model 2: all variables included. Multivariable analysis was performed using logistic regression and results were expressed as odds ratios and 95% confidence intervals

Restricting the analysis to the WTD as assessed either by the SAHD-senior or the CADO produced the same results, except that age was no longer significantly associated with the WTD assessed using the SAHD-senior (Additional Table 2).

### Degree of stress

The median and [interquartile range] of the reported stress score was 0 [0–1] and 0 [0–0] for patients with and without a WTD, respectively (Mann–Whitney test, *P* = .102).

## Discussion

We report for the first time the prevalence of the WTD in elderly patients hospitalized for acute care in internal medicine. One out of twelve patients expressed a WTD, and QoL was the main determinant of the WTD. Our hypotheses were therefore only partially supported.

### Prevalence of the wish to die

Prevalence of the WTD was low: 5.6% according to SAHD-senior scores, 6.5% according to CADO scores, and 8.6% according to both scores. Previous studies of older adults in non-acute hospital settings showed a prevalence of 8.9–14.9% (using either or both instruments) in geriatric rehabilitation inpatients [3], and 4.0–22.1% (using either or both instruments) in nursing homes [4]. WTD prevalence in this study was also lower than for palliative care patients in a previous study using the SAHD, which found a prevalence of between 3.9 and 28% (4.6 to 17% using the same cutoff as in the present study) [30]. A likely explanation for the lower WTD prevalence in our study is that elderly patients hospitalized in acute care expect a favourable outcome and do not consider death an option.

Of the 20 patients expressing a WTD according to SAHD-senior or CADO scores, only 8 (40%) were identified by both instruments. Indeed, there was a moderate agreement between instruments, suggesting that they cannot be used interchangeably. This moderate agreement may explain the different prevalence rates and associations between WTD and other covariates, as the instruments do not capture the WTD in the same patients. This variability between WTD instruments has been noted previously [30]. The authors of a previous validation article found lower variation between the SAHD-senior and the CADO [3], perhaps because of the non-acute context, where patients present with more chronic symptomatology. Overall, our results suggest that WTD prevalence is low but highly relevant for clinical practice in an acute care setting, and that the two WTD instruments do not capture the WTD in the same individuals.

### Determinants of the wish to die

Older age was associated with a greater likelihood of the WTD, a finding also reported previously [4, 16]. The association between older age and the WTD was particularly strong in the present study, probably because the average age was almost 80 years. According to one systematic review, most other studies have assessed patients with an average age of 60 years [30]. The fact that elderly people are more accepting of death [31] may explain the strong link between the WTD and age in our study. Importantly, most elderly participants had a passive WTD. This suggests that elderly patients do not want active interventions to induce the end of life, but wish for death as a consequence of the disease course. This is consistent with findings from a study on end-of-life decisions among 38 chronically ill Canadian elderly inpatients: most patients rejected the idea of active interventions to induce death, but favoured the withholding and withdrawing of treatment [32].

Worse QoL was associated with a greater likelihood of the WTD. This association was in line with our initial hypothesis and, to our knowledge, no other studies have assessed the association between QoL and WTD. Interestingly, no association was found between other biopsychosocial–spiritual factors and the WTD, probably because QoL surpasses other biopsychosocial–spiritual variables as a reason for (not) wishing to die. Indeed, it has been shown that elderly persons with significant limitations or illnesses may rate their QoL as excellent, a condition termed the ‘disability paradox’. This is because QoL cannot be reduced to a general health status, and includes psychological resources or family support [33], as assessed in this study. Therefore, patients who consider their QoL as adequate may not express a WTD, and interventions that positively influence QoL may limit the WTD.

Presence of depressive symptoms was associated with a higher WTD in the bivariate analysis, but this association was no longer significant after multivariable adjustment. Our findings do not replicate those of previous studies [7], and could be explained by the fact that medically ill elderly patients score higher on the CES-D somatic items regardless of their psychological status [34]. Indeed, we found that 23% of patients without a WTD had clinical depression, compared with only 9% in a study of community-dwelling Dutch elderly people [2]. Overall, our results suggest that depression may not be as important as QoL in influencing the WTD among elderly hospitalized patients.

Prevalence of DNR orders was higher than reported in a previous study (71% vs. 53%) [13]. This may be because our sample was older, the likelihood of a DNR order increases with age, and the systematic collection of DNR orders has been progressively implemented in our hospital since 2013. No significant association was found between the WTD and DNR orders. A possible explanation is that participants are aware of the inevitability of their death, but still hope to recover from an acute illness and leave hospital alive. Indeed, a previous study showed that preference to die at home was associated with higher odds (8.29) of DNR orders [35]. As no information related to place of death was collected, we cannot confirm this finding. This absence of significant association is consistent with a previous study, among end-stage amyotrophic lateral sclerosis patients [8].

### Degree of stress

Participants did not experience stress during the interview. The safe environment, which included a period of patient listening, may have had a positive effect on participants. These findings are consistent with those from studies of end-of-life patients [36, 37], and suggest that WTD assessment, when adequately implemented, does not affect the mood of participants. Hence, this topic should not be avoided by care teams when directly brought up by the patient [36, 38]; furthermore, these types of discussions can be initiated by clinicians.

### Implications for clinical practice and research

Our results provide useful information for future studies assessing the prevalence and determinants of the WTD. First, there should be no anxiety about discussing the WTD with patients. Second, SAHD-senior and/or CADO assessments can be used in internal medicine wards, where we have described a significant prevalence of WTD. The CADO is quickly administered and can be used as a screening tool. In contrast, the SAHD-senior may help to identify the intensity of a WTD [3]. Ideally, the WTD should be assessed several times during the patient’s stay, given its evolution over time [39]. Third, QoL can be assessed with a single question rather than with complex questionnaires.

### Study limitations

This study has some limitations. First, we pooled the results from two instruments measuring different WTD dimensions. However, this is common practice [4] and the conclusions were similar when the analyses were restricted to each instrument. Second, the number of patients expressing a WTD was low, which reduces the statistical power of the analysis. However, compared with other studies, our sample size can be considered large. Only 93% of the calculated sample size was reached, which may have also slightly reduced the statistical power. However, we believe that the magnitude of this reduction was minimal. Third, we cannot exclude the possibility of selection bias; patients who were more depressed or more unwell may have refused to participate because they could not engage with this sensitive issue. Hence, it is possible that WTD prevalence may have been underestimated and that some associations (e.g. between QoL and the WTD) may be stronger than reported. Further, owing to consent issues, it was not fully possible to compare the characteristics of our sample with those of similar hospitalized patients. Hence, we cannot assume that our sample is representative of the target population. Fourth, the cross-sectional design of the study cannot take into account the fluctuating nature of the WTD [39]. However, many studies have assessed WTD only once [2–4, 16, 17], as the variability of the WTD would add an extra complexity to the data analysis and interpretation. Finally, the study was conducted at a single hospital in French-speaking Switzerland, which has a population characterized by a high prevalence of atheism [40] and a high educational background. Unlike in many other countries, legalized assisted suicide is available in Switzerland, which facilitates the discussion of death among the population. Hence, our results may not be generalizable to other countries. It would be of interest to replicate our study in such countries and to compare the results.

## Conclusions

WTD prevalence among elderly patients admitted to an acute hospital setting was low, but highly relevant for clinical practice. The agreement between two instruments assessing the WTD was moderate. Older age increased and better QoL reduced the likelihood of the WTD. Discussion of death appeared to be well tolerated by patients. More research on how to assess WTD is needed.

## Supplementary information

**Additional file 1.**

## Data Availability

Individual data from participants who consented for their data to be shared with other investigators can be made available. Requests will be evaluated on an individual basis and, if necessary, submitted to the ethics commission of Canton Vaud for approval.

## Abbreviations

CADO: Categories of Attitudes toward Death Occurrence
CASP-12: Quality of life – Control, Autonomy, Self-realization and Pleasure
CES-D: Center for Epidemiologic Studies – Depression
DNR: Do not resuscitate
MOS-SSS: Medical Outcome Study – Social Support Survey
QoL: Quality of life
SAHD-senior: Schedule of Attitudes toward Hastened Death – senior
SPIR: Spiritual needs and preferences
WTD: Wish to die
