# Supplementary material for: Life worth living: cross-sectional study on the prevalence and determinants of the wish to die in elderly patients hospitalized in an internal medicine ward
Source: BMC Geriatr. 2020 Sep 14;20:348. doi: 10.1186/s12877-020-01762-x (PMC7491164; doi:10.1186/s12877-020-01762-x)
Supplement: Supplementary file 1 — Additional file 1. [file 12877_2020_1762_MOESM1_ESM.docx]

**ADDITIONAL TABLES**

*Additional table 1.* Characteristics of participants who did and who did not consent to participate in the study.

|  | **Did not consent** | **Consented** | ***P*-value** |
| --- | --- | --- | --- |
| Sample size | 89 ^a^ | 232 |  |
| Age (SD), years | 79.3 (7.1) | 79.3 (8.1) | .989 |
| Women (%) | 42.7 | 44.8 | .731 |
| Charlson score (SD) | 10.1 (16.7) | 5.9 (11.0) | .017^b^ |
| Number of medications (SD) | 7.7 (4.4) | 7.4 (3.8) | .658^b^ |
| Functional status (SD) | 6.3 (1.2) | 6.3 (1.3) | .882 |
| DNR order (%) | 71.6 | 71.4 | .977 |

DNR, do not resuscitate. ^a^ 307 patients did not consent, but data was available for 89 only: owing to Swiss legislation regarding research involving human beings, it was not possible to collect information from the medical records of patients who refused to participate and who did not consent to share their medical data. Results are expressed as average (standard deviation) for continuous variables, or percentage for categorical variables. Between-group comparisons were performed using student’s t-test or Mann-Whitney test (^b^) for continuous variables and chi-square for categorical variables.

*Additional table 2.* Multivariable analysis of the determinants of the wish to die, using SAHD-senior or CADO only.

|  | **Model 1** | ***P*-value** | **Model 2** | ***P*-value** |
| --- | --- | --- | --- | --- |
| **SAHD-senior** |  |  |  |  |
| Age (per 5-year increase) | - |  | 1.25 (0.83 - 1.89) | .292 |
| CASP-12 (per 5-point increase) | 0.35 (0.20 - 0.62) | <.001 | 0.73 (0.34 - 1.55) | .411 |
| QoL - Single question | 0.56 (0.43 - 0.75) | <.001 | 0.64 (0.45 - 0.90) | .012 |
| CES-D (per 2-point increase) | 1.26 (1.09 - 1.46) | .002 | 1.11 (0.92 - 1.34) | .270 |
| **CADO** |  |  |  |  |
| Age (per 5-year increase) | - |  | 1.68 (1.08 - 2.61) | .022 |
| CASP-12 (per 5-point increase) | 0.35 (0.20 - 0.62) | <.001 | 0.72 (0.34 - 1.51) | .382 |
| QoL - Single question | 0.54 (0.41 - 0.73) | <.001 | 0.55 (0.38 - 0.79) | .001 |
| CES-D (per 2-point increase) | 1.16 (0.99 - 1.37) | .061 | 0.98 (0.78 - 1.21) | .825 |

CADO, Categories of Attitudes toward Death Occurrence; CASP-12, Control, autonomy, self-realization and pleasure 12 items – French version; CES-D, Center for epidemiologic studies – depression; QoL, Quality of life; SAHD-senior, Schedule of Attitudes toward Hastened Death – senior. Model 1: adjusted for age; model 2: all variables included. Multivariable analysis was performed using logistic regression and the results were expressed as odds ratio and 95% confidence interval.
